# First Rank Symptoms and Neurological Soft Signs in Schizophrenia

**DOI:** 10.1155/2014/931014

**Published:** 2014-02-13

**Authors:** Mahesh Hembram, Jayati Simlai, Suprakash Chaudhury, Parthasarathi Biswas

**Affiliations:** ^1^Department of Psychiatry, Ranchi Institute of Neuropsychiatry and Allied Sciences, Ranchi, Jharkhand 834006, India; ^2^Department of Psychiatry, Rural Medical College & Hospital, Pravara Institute of Medical Sciences (Deemed University), District Ahmednagar, Loni, Maharashtra 413736, India

## Abstract

The aim of the study was to compare the neurological soft signs (NSS) in schizophrenia patients with and without first rank symptoms (FRS), their first degree relatives (FDR), and normal controls. The study was conducted on 60 schizophrenia patients diagnosed according to ICD 10 DCR and categorized into groups with and without FRS using Schedules for Clinical Assessment in Neuropsychiatry, 30 FDRs of the study sample, and 30 normal controls matched for age, education, and handedness. All the subjects gave written informed consent. Scale for the Assessment of Positive Symptoms and Scale for the Assessment of Negative Symptoms were applied to have a comprehensive assessment of the symptoms. NSS were assessed using Extended Standard Neurological Assessment Instrument. The correlations between NSS and clinical symptoms were relatively modest but significant. There was a weak relation between NSS and positive symptom severity. The FDR of schizophrenia patients had significantly lower NSS scores than schizophrenia patients, but only FDR of schizophrenia patients without FRS had significantly higher scores than normal controls. Our results indicate that NSS are more prominent in schizophrenia patients with negative symptoms and support the theory of NSS being a trait marker of schizophrenia particularly in those without FRS.

## 1. Introduction

A prominent conceptualization of schizophrenia is as a neurodevelopmental disorder, where genes and environment interact over the course of development to determine abnormalities in neural systems that give rise to the disorder. As the brain matures through childhood, the illness is further expressed, ultimately manifesting in late adolescence and adulthood as psychotic symptomatology [1, 2]. Kurt Schneider, a German psychiatrist and a pupil of Karl Jaspers, pointed out certain symptoms as being characteristic of schizophrenia and therefore exhibiting a “first-rank” status in the hierarchy of potentially diagnostic symptoms [3]. The “first-rank” symptoms (FRS) are a group of intriguing experience characterized by striking breach of “self versus non-self” boundaries. FRS have played an extremely important role in the recent diagnostic systems: in the International Statistical Classification of Diseases, Tenth Revision (ICD-10) [4], as well as in Diagnostic and Statistical Manual of Mental Disorder, Fourth Edition (DSM IV) [5]. The genesis of FRS can be understood in the paradigm of “abnormalities in the awareness of action” [6]. The first rank symptoms included audible thoughts, voices arguing, voices commenting, delusional perception, somatic passivity, made affect, made impulses, made volition, thought insertion, thought withdrawal, and thought broadcasting [7]. Elucidating the pathophysiology of FRS is of great interest, not only because these symptoms are the hallmark of psychotic “alienation,” but because also recent studies have reported that FRS had familial characteristics, suggesting that heritable factors for psychosis vulnerability may be implicated in the brain dysregulation favouring the emergence of FRS [8, 9].

Neurological abnormalities are traditionally classified as “hard signs,” impairments in basic motor, sensory, and reflex behaviors, which do not appear to be affected in schizophrenia, and “soft signs,” which refer to more complex phenomena such as abnormalities in motor control, integrative sensory functions, sensorimotor integration, and cerebral laterality [10]. Neurological soft signs (NSS) are neither indicative of dysfunction of a specific brain region nor part of a well-defined neurological syndrome. A correlation between NSS, the general severity of psychopathology, and positive schizophrenic signs in first episode schizophrenic patients was reported [11], but other authors have not corroborated the association of NSS with either the general level of psychopathology [12] or the positive or negative dimension of schizophrenia [13]. However, several lines of research indicate a relationship between NSS and the pathophysiology of schizophrenia. In comparison to controls, higher levels of NSS have been noted in first-episode [14], as well as both medicated and treatment-naive individuals with schizophrenia [15]. Rates of NSS are also elevated in individuals at high risk for schizophrenia compared to controls [16]. Furthermore, there is evidence for a genetic component to NSS, as family members of schizophrenia patients exhibit higher levels of NSS than matched control subjects [17]. Several studies have examined the relationship between NSS and schizophrenia symptoms; however, results have been equivocal. While one study found a correlationship between NSS and positive symptomatology [11], other studies did not detect an association [13, 18]. Similarly, one study found an association between NSS and negative symptoms [19], while others have found no such relationship [20, 21]. There is some evidence indicating that NSS may be related to response to antipsychotic treatment in schizophrenia patients. One study, focusing on a first-episode sample, noted that an improvement in positive symptoms six weeks after commencing antipsychotic treatment was positively correlated with improvement in NSS [8]. In another sample of first-episode schizophrenia patients, researchers found that six months after the initial episode, improvement in total NSS was positively correlated with improvement in positive symptoms [9].

Neurological soft signs have also been found with increased frequency in relatives of those with schizophrenia. An increased prevalence of abnormal neurological signs (ANS) in chronic schizophrenics as compared to acute schizophrenics has been reported [22]. This may be explained by a number of considerations. First, chronic schizophrenics have had a prolonged course. Increased ANS in these subjects may be a marker for chronicity and severity of the illness. Second, chronic patients potentially have been exposed to more antipsychotic medications, which may increase the presence and severity of ANS. Third, the severity of ANS may progress with the course of the illness independently of other factors. The presence of ANS in schizophrenia can in part be explained by a neurodevelopmental theory for the illness. Previous reports have proposed that these neurodevelopmental abnormalities may play a role either causative or additive in the pathogenesis of schizophrenia [23]. This theory is consonant with speculation that children at risk for developing schizophrenia show evidence of neurointegrative defects [24].

Only a few studies have assessed the relation between psychopathology and NSS in a sufficient number of patients [25]. NSS are classified by some authors as the trait markers of schizophrenia, while others consider them to be state markers [12, 26]. The occurrence of NSS in the initial stage of the disease and especially the fact that they occur although less often in healthy relatives of patients with schizophrenia indicated their inclusion among trait markers [11, 14]. On the other hand, their variable intensity over the course of the disease which relates to a clinical course and especially to recovery rate is indicative of state markers [27]. It is against this background that the present investigation was carried out to determine the occurrence of NSS in patients with schizophrenia. Deepening our understanding of NSS in schizophrenia may help to elucidate the pathophysiology of this disorder as well as improve our ability to successfully treat schizophrenia patients. The aim of this open, naturalistic study was to examine the relationship between NSS and FRS in patients with schizophrenia, their first degree relatives, and normal control.

## 2. Materials and Methods

This study was carried out with inpatients having schizophrenia at Ranchi Institute of Neuropsychiatry and Allied Sciences. This is a referral center for all acute psychiatric hospitalizations and outdoor patients within its catchment area which includes patients from states of Jharkhand, Bihar, Orissa, Chhattisgarh, and West Bengal. The protocol for the study was submitted to and approved by the institutional ethical committee.

## 3. Study Design and Sample

The subjects for this cross-sectional study were recruited by purposive sampling technique. The experimental group consisted of 60 drug-free or drug-naïve schizophrenia subjects diagnosed clinically according to the ICD 10 DCR [28] criteria for schizophrenia. The sample was divided into two groups of patients of schizophrenia with and without FRS (30 patients each group). All patients were in the age range of 18 years to 60 years with onset psychosis after the age of 18 years. Due to administrative reasons, only male patients could be included. The patients were drug-naïve or drug-free (drug-free being defined as being off oral antipsychotic medications for a period of 4 weeks and for long-acting antipsychotic a period of 8 weeks). Exclusion criteria included patients with serious medical disorder, neurological condition, head injury, epilepsy, and substance use disorder (within 6 months of assessment, but excluded patients with a score of ≤6 in Drug Abuse Screening Test). Patients with Mini-Mental Status Examination score of <24 were excluded and so were patients with an independent diagnosis of mood disorder or history of psychiatric disorder or mental retardation. For control, 30 first degree relatives of the patients of the study sample were taken. Normal controls matched for age, education, and handedness were also inducted in the study. All the subjects gave written informed consent.

## 4. Procedure for Data Collection and Tools Used

Patients meeting the inclusion and exclusion criteria were assessed in detail on the semistructured sociodemographic data sheet devised for the study. Initial screening was done to rule out dementia or any cognitive impairment using the Mini-Mental status Examination (MMSE) [29] and significant substance use using the Drug Abuse Screening Test (DAST) [30]. Patients were evaluated for handedness using the Edinburgh Handedness Inventory (EHI) [31].

FRS (voices commenting or discussing, delusion that thoughts are being read, loud thoughts, thought echo, thought insertion, thought broadcast, thought commentary, thought block, thought withdrawal, replacement of will by external force (somatic passivity), replaced control of actions, replaced control of affect, other experiences of replaced control (impulse), and delusional perception) were assessed using items from the Schedule for Clinical Assessment in Neuropsychiatry (SCAN), Version 2.1 [32]. SCAN is a set of tools created by WHO aimed at diagnosing and measuring mental illness that may occur in adult life. It is not constructed explicitly for use with either ICD-10 or DSM-IV but can be used for both systems. The entire SCAN interview consists of 1,872 items, spread out over 28 sections. Studies assessing the prevalence of FRS have used items from SCAN [33]. FRS was obtained by summing the individual global item scores [34, 35]. From the SAPS, a first rank symptom score was obtained by summing the scores of six items [34, 35]. Additionally, from all of the available information, interviewers were asked to rate the presence or absence of the FRS.

Scale for the Assessment of Positive Symptoms (SAPS) [36] was used to evaluate positive symptoms which include hallucinations, delusions, bizarre behavior, and formal thought disorder. It is used in conjunction with Scale for the Assessment of Negative Symptoms (SANS) [37] to have acomprehensive assessment of the symptoms.

Soft neurological signs were assessed using Extended Standard Neurological Assessment Instrument [38–40]. This instrument has 44 items, which encompasses the sensory, motor, reflexes, and cognitive domains. Both hard and soft signs have been incorporated in this instrument. Interrater reliability for the neurological assessment between the examiner and two other physicians was (intraclass correlation) 0.87 and 0.97, respectively (*P* < 0.001) [38]. A few small modifications were done to make the instrument suitable for the Indian patients (e.g., in item no. 20, tying a rope in males or tying their hair in females instead of tying shoelace as most of the patients did not wear shoes). The patient's insight was assessed using Scale to Assess Unawareness of Mental Disorder (SUMD) [41]. Global assessment functioning (GAF) [5] scale was also applied while interviewing the informants.

## 5. Data Processing and Analysis

Data was processed using Statistical Package of Social Sciences—version 16.0 (SPSS-16). Descriptive statistics was used to calculate mean, percentage, and standard deviation of the sample. Chi-square and one-sample *t*-test were used to compare the mean values between patients with and without FRS. Spearman Rho was used to find the correlation. Regression analyses were used to determine parameter of continuous variables. The relationship between variable was computed using ANOVA. The level of significance was kept at *P* < 0.05 (2-tailed).

## 6. Results

### 6.1. Sample Characteristics

#### 6.1.1. Sociodemographic Characteristics

All the patients of schizophrenia with and without FRS, their first degree relatives (FDR), and normal control were males. The sociodemographic characteristics of the sample (Table 1) show the comparison of age and income between patients of schizophrenia with and without FRS, their FDR, and normal control. There is a no significant difference in age among the groups. Normal controls and FDR of patients of schizophrenia with and without FRS had significantly greater income as compared to the study population.

The comparison of residence and employment status between patients of schizophrenia with and without FRS (Table 2) did not differ in the demographics, residence, religion, ethnicity, and marital status. There was a significant difference in employment status. Rate of unemployment was greater (*n* = 17, 56.7%) in patients of schizophrenia without FRS. Patients of schizophrenia with FRS showed higher percentage of meaningful employment (*n* = 16, 533.3%).

Table 3 shows the comparison of age of onset of psychosis and duration of untreated psychosis between patients of schizophrenia with and without FRS. There was no significant difference in the two groups on these clinical variables. Results of comparison of SAPS scores between patients of schizophrenia with and without FRS show statistically significant difference in global rating of severity of hallucinations, global rating of formal thought disorder, and total SAPS score (Table 3). However, there was no significant difference in global rating of delusion and global rating of bizarre behavior. Comparison of SANS scores between patients of schizophrenia with and without FRS shows high significant difference in global rating of attention and total SANS score (Table 3). The other domains of SANS did not show any significant difference.

The frequency of the FRS in patients of schizophrenia showed that voices commenting was the most commonly reported symptom (*n* = 21; 70%), followed by thought broadcasting (*n* = 10; 33.3%), thought insertion (*n* = 8; 26.7%), replaced control of actions (*n* = 7; 23.6%), delusions that thought are being read (*n* = 4; 13.3%), and thought withdrawal (*n* = 4; 13.3%). Replacement of will by external force (somatic passivity), replaced control of affect, and delusional perception were each reported by 2 (6.7%) patients. Thought echo, thought commentary, thought block, and other experiences of replaced control (impulse) were each reported by 1 (3.3%) patient.

Table 4 shows the comparison of mean rank scores of NSS between patients of schizophrenia with and without FRS using Kruskal-Wallis test. The degree of freedom (df) is 4. Table 4 also shows the post hoc comparison between the groups. The critical value calculated was 10.5112. The table shows that schizophrenia patients (with and without FRS) scored significantly higher in total abnormality score, filtered abnormality score, soft signs, hard signs, cranial nerves, cognitive functions, reflexes, and sensory scores. Filtered hard signs, motor domain, involuntary movements, and muscle power did not show any significant differences. FDR of schizophrenia patients scored significantly less than schizophrenia patients in total abnormality score, filtered abnormality score, soft signs, and hard signs. However, only FDR of schizophrenia patients without FRS scored significantly higher than normal controls on total abnormality score, filtered abnormality score, and soft signs.

Table 5 shows significant positive correlation between reflex and total SANS score (rho = 0.35, *P* = 0.006, two-tailed). There was no other significant correlation between total abnormality score (0–125), soft signs, mirror movements, cranial nerves, Domain II: cognitive functions, Domain IV: sensory, and total SANS score.

There was no significant correlation between total abnormality score (0–125), soft signs, mirror movements, cranial nerves, Domain II: cognitive functions, Domain III: reflexes, Domain IV: sensory, and total SAPS score.

Table 6 is a linear regression model of demographic and clinical variables of schizophrenia with and without FRS. Using the enter method, a significant model emerged (*F* = 2.62, df = 7.22, and *P* = 0.040) Adjusted *R* Square = 0.281. The dependent variable was motor I domain. Age of onset of psychosis (Beta = 4.276, *P* = 0.042) and total SAPS score (Beta = −0.46, *P* = 0.047) were significant at the level of 0.05, two-tailed. Total SANS score (Beta = 0.76,  *P* = 0.001) was highly significant at the level of 0.001, two-tailed. The negative value of Beta for SAPS indicates that NSS decrease as SAPS scores increase.

## 7. Discussion

The present study was driven by the renewed interest in the construct of the FRS which represents an important leakage in self-perception and should be understood as a symptom of ego disturbance or an invasion of the boundaries of self [42]. This could explain why FRS are considered by some as signs of severe psychopathology. This led to hypothesize that the presence of FRS predicts a poorer outcome in schizophrenia [43] and in other psychosis.

### 7.1. Selection of the Sample

Stringent criterion for selection of the patients was kept to avoid any contamination of the samples. First and foremost patients diagnosed with schizophrenia and related disorders were screened out for presence or absence of FRS. Patients either having FRS or not having FRS were given an MMSE to rule out cognitive deficits due to dementia. However, there are a number of limitations that have emerged with widespread usage of the MMSE. Patients with high premorbid intelligence or education show a ceiling effect, thus leading to false negatives. Great age, limited education, foreign culture, and sensory impairment can produce false positives. Consequently, MMSE score needs adjustment for age and education. The patients with substance dependence syndrome or using substance in harmful pattern were exempted from the study employing the Drug Abuse Screening Test. Using cut-off score of <11 somewhat reduces the sensitivity for identifying patients with substance use disorder and more accurately identifies the patients who do not have substance use disorder. Care was also taken to rule out patients currently using any oral antipsychotic medication in the last 4 weeks and 6 weeks of long-acting injectable (depot) antipsychotic medication to wash out the effect of the drugs. An attempt was also made to take patients with recent onset of illness but due to the stringent criterion exceptions had to be made to complete the study.

### 7.2. Discussion of Sociodemographic Characteristics

Our study included patients in the age range of 18–60 years. The minimum age was kept above 18 to avoid accidental inclusion of patients with developmental delays. The maximum cut-off age was kept below 60 years to avoid inclusion of patients with age-related neurological or cognitive changes. The comparison of age in patients of schizophrenia with and without FRS, their FDR, and normal control did not show any significant difference between the groups. This was similar to the findings of earlier studies [44]. In the Danish OPUS study of 362 young adult patients with first-episode schizophrenia, also there was no significant difference in the mean age of schizophrenia patients with and without FRS [45].

Neurodevelopmental aberrations can lead to dysregulated connectivity between the hemispheres [46], and, indeed, deficient interhemispheric transfer has been documented in schizophrenia (hypoconnectivity hypothesis) [47]. Interestingly, hyperconnectivity has been postulated to underlie the genesis of Schneider's FRS, as supported indirectly by the functional imaging studies which have implicated hyperactivation of right-parietal area as neuroanatomical substrates of FRS [48, 49]. Also, when normal controls were led to believe that another person was controlling their actions, there was activation of the inferior parietal lobule [50]. Thus, FRS in schizophrenia are associated with hyperactivity in the parietal cortex [50, 51]. Fibers from parietal cortex implicated in FRS will traverse in the splenium of corpus callosum [47]. A recent study suggests that those with FRS had larger splenium than those without FRS supporting the hyperconnectivity hypothesis [52]. Also, connectivity abnormalities might be reflected by the age at onset, as observed in a recent study, in which there was a significant positive correlation between age at onset and FRS score. That is, those with FRS had later age at onset than those without FRS [52]. In the present study, also the mean age of schizophrenia with FRS was higher than those without FRS though the difference was not significant. Another study found that cognitively complex FRS did not appear before adolescence and suggested that a certain degree of cognitive development is necessary to display such experiences [53]. Interestingly, one factor that influences the clinical manifestations of any cerebral lesion is the age of the brain. In fact, the etiology of schizophrenia has focused on postpubertal brain changes that may be involved in triggering the expression of vulnerability for abnormal brain development [54].

In a study of neurological abnormalities (NAs) in schizophrenic twins, NAs were increased in probands with schizophrenia compared to nonschizophrenic cotwins and to healthy control twins but there were no significant differences between patients from the concordant and discordant pairs. NAs in the nonpsychotic cotwins from discordant pairs were increased compared to control twins [55]. These findings implicate genetic factors, in determining the level of NA detected in the well cotwins of patients with schizophrenia. Results indicated that relatives demonstrate a similar distribution of deficits to probands and that the magnitude of those deficits is determined at least in part by the degree of genetic risk. The findings imply that NAs are a robust finding in schizophrenia, determined in part by the inherited genetic risk for the disorder. A number of other studies have used the same scale [38] which was used in this study. The results of these previous studies show that patients with schizophrenia and their siblings score higher than normal controls on the soft signs total, as well as the sensory integration and motor functioning subscales. The other variables like residence, religion, ethnicity, and marital status between patients of schizophrenia with and without FRS did not show any significance. Previous studies also have not shown a significant difference in the sociodemographic profiles [22, 45, 56]. Table 1 shows the comparison of income between patients of schizophrenia with and without FRS, their FDR, and normal control with regard to income. The lower income of patient without FRS could be explained from the fact that this group had an earlier age of onset of schizophrenia thereby limiting their working capacity and also had higher levels of unemployment.

### 7.3. Discussion of the Clinical Characteristics

Clinical variables like age of onset of psychosis and duration of untreated psychosis between patients of schizophrenia with and without FRS did not show any significant difference in the two groups (Table 3). However, in an earlier study of 100 schizophrenia patients, 34 (22 males and 12 females) reported having one or more FRS. These patients were older in age and married, whereas the illness was of chronic nature [56]. Although there was no significant difference in the level of neurological soft signs at initial presentation, after clinical stabilization, the level motor soft signs were significantly lower in patients with a shorter DUP. The study showed that patients with a short DUP have lower levels of neurological soft signs at the clinical stabilization stage [22]. Results from the Danish OPUS study show that in first-episode schizophrenia patients the duration of untreated psychosis and the age of onset of psychosis did not differ significantly in schizophrenia with and without FRS. However, there was a significant correlation between the psychotic symptom score and FRS [45].

The present study shows a significant difference between patients of schizophrenia with and without FRS in global rating of severity of hallucinations (Table 3). The table also shows significance between patients of schizophrenia with and without FRS in areas of global rating of formal thought disorder (FTD), total SAPS score, global rating of attention, and total SANS score. This finding is in agreement with the Danish OPUS study [45]. The current study shows (Table 5) significant positive correlation between reflex and total SANS score. However, being the only finding, its occurrence by chance cannot be ruled out.

Voices commenting was the most commonly reported symptom (70%) in our study, followed by thought broadcasting (33.3%). Replaced control of actions was next with 23.6%. Delusion that thoughts are being read and thought withdrawal occurred in 13.3% of the patients. Loud thoughts did not occur in any of the study samples. Our findings are partly in agreement with an earlier study [45] which reported that almost half of the patients had experienced commenting or discussing voices, and more than 40% had experienced loud thoughts. Seventy-seven percent of the patients reported frequent or severe FRS, which indicates that FRS are very common among patients with first-episode schizophrenia. Mellor [57] also reported that 72% of a group of patients with schizophrenia had FRS, although not all patients were first-episode in that sample. Two other studies found the prevalence of FRS in schizophrenia to be 73% and 70%, respectively [58, 59]. These results underline the relevance and the importance of FRS as a part of the diagnostic criteria although they are not as specific to schizophrenia as Schneider suggested.

Comparison of soft neurological sign in patients with positive and negative schizophrenia revealed that the total score of the Neurological Evaluation Scale (NES) was significantly higher in the negative subtype group, indicating higher neurological impairment in the patients with negative symptoms [60]. This finding is in agreement with the results of logistic regression in the present study (Table 6). Relationships between neurological signs and different clinical symptoms are not a consistent finding. For example, Tiryaki et al. [61] found that the subscore of sequencing of complex motor acts is a significant predictor of deficit state, which is the negative subtype of schizophrenia. While there was no correlation either between the PANSS scale for positive symptoms and the total PANSS score or the scores for disorganization and reality distortion syndromes and any NES score, it was found that the PANSS scale for negative symptoms score correlated positively with the “others” NES subscore and that the psychomotor poverty syndrome score correlated positively with the “others” subscore and the total score of NES [25].

### 7.4. Discussion of the Diagnostic Specificity of FRS

Examining Schneiderian FRS is associated with several problems at the practical level as well as at a more overarching epistemological level. A fundamental issue in the reviewed studies is the question of validity of psychiatric diagnosis in general and of schizophrenia in particular. There is a lack of explicit realization that the diagnosis of schizophrenia is based on a certain convention and not on its purported essential nature. Unclear definitions of the FRS are another widespread problem in the studies; neither Schneider nor Present State Examination (PSE) offers very precise definitions of FRS. Consequently, different explications of FRS are possible, and if the precise definitions are not articulated, it is difficult to compare the conclusions of the studies. This confusion is also found in the ICD-10. Here, the diagnoses are ordered hierarchically and schizophrenia precedes affective disorder in the classification. The FRS are specified in the operational criteria as being of special importance for the diagnosis of schizophrenia. Yet, it is stated that “the diagnosis of schizophrenia should not be made in the presence of extensive, depressive, or manic symptoms unless it is clear that schizophrenic symptoms antedated the affective disturbance.” This undermines the hierarchical structure of the system and, even more importantly (as was the case with the DSM-IV), it deprives the clinician of the support, implicitly intended, from the symptoms of specific importance like the FRS. However, in 40% of the patients, 3 of the 10 most frequent subjective symptoms antedating schizophrenia are affective: restlessness, depression, and anxiety, and many qualify for a full depressive syndrome [62, 63]. Should these patients be diagnosed as depressed for the rest of their lives? It is also essential to clarify how we conceive the FRS, what their phenomenological nature is, and what method is adequate to assess their presence and diagnostic importance. Some critics noted that the FRS were only diagnostic of schizophrenia on the condition of a simultaneous “phenomenological leverage” [64]. The “phenomenological leverage” was mentioned by Schneider himself, albeit in a rather lateral and cryptic manner: “(the FRS) signify a radical qualitative change in the thought processes as described by Gruhle.” In summary, Schneider, his contemporaries, and more recent phenomenological contributions [65, 66] did not consider the FRS as atomic symptoms but as two groups of phenomena: passivity experiences and hallucinations, with certain phenomenological overlaps [8]. A call for a “phenomenological leverage” points to several problematic aspects of their diagnostic role, of which we will mention only one. Thus, the FRS are diagnostically useful only in a patient without clouding or other degradation of consciousness (e.g., with a perplexity due to severe emotional turmoil, strong fear or anxiety, extreme mood, or psychomotor swings) simply because the trait-like, formal alterations of experience and consciousness can only be meaningfully assessed in a patient whose consciousness is in a state of “composure” [3].

### 7.5. Neurological Soft Signs (NSS)

In the present study, schizophrenia patients with FRS had a greater mean rank in total abnormality score, filtered abnormality score, soft signs, and hard signs followed by patients without FRS (Table 4). Within group comparison shows FDR mixed results. The FDR of schizophrenia patients had significantly lower NSS scores than schizophrenia patients, but only FDR of schizophrenia patients without FRS had significantly more scores than normal controls (Table 4). Findings are consistent literature showing a graded pattern of NSS severity, with healthy relatives having an intermediate number between patients and controls [67].

In our literature search results, three studies indicated a significant correlation for NSS scores between schizophrenia patients and their relatives, but only one study reported on the heritability of NSS in schizophrenia [68]. With regard to the state-independent criterion, the existing literature suggests that NSS have been demonstrated in schizophrenia at different stages of the illness [22, 27, 69]. According to the previous review, NSS occur more frequently in schizophrenia patients than healthy controls at all stages of the illness [70]. However, most of the existing evidence is limited to cross-sectional studies.

Most of the studies have used the NES in samples of patients with schizophrenia; some produced results that support the data from the current study. Ismail et al. [71] reported that patients with schizophrenia and their siblings scored higher than normal controls on the Soft Signs Total, as well as the Sensory Integration and Motor Functioning subscales. Additionally, patients with schizophrenia have been reported to score higher than at-risk patients, who in turn scored higher than controls on the soft signs total, sensory integration, and other soft signs [72]. Arango et al. [73] reported that the Other Soft Signs subscale was able to correctly classify a greater number of patients and controls to their true group than the other subscales from the NES. Taken together these previous studies and the current results suggest that the other soft signs subscale may be particularly sensitive in identifying those with schizophrenia or a proneness to it.

Griffiths et al. [74] suggested that the presence of soft signs in relatives increases with the potential genetic loading (i.e., greater incidence of schizophrenia in a family increases the presence of soft signs). Furthermore, Gourion et al. [17] reported that the total soft signs score could be used to distinguish relatives who were thought to be carriers of the genetic vulnerability to schizophrenia from those who were not. This suggests that the presence of neurological soft signs may be indicative of being a “gene-carrier” for psychosis. It is not possible, on the information available, to determine whether the participants in the current study are gene-carriers for schizophrenia. However, the results from Lawrie et al. [72] may suggest that soft signs are not an indicator of genetic risk specifically for psychosis. Other causes of soft signs may be low birth weight [75] and obstetric complications [76]. Obstetric complications may be a more significant factor for males at risk for schizophrenia or for leading to soft signs in those at genetic risk for developing schizophrenia [77].

NSS have been associated with a more severe clinical course of psychosis, worse psychosocial performance, and cognitive dysfunction [18, 78–80], suggesting that they may characterize a subgroup of patients sharing a more severe pathophysiological process. The comparison of NSS rates in patients with psychosis across different studies is made difficult by the fact that they are evaluated with a variety of instruments and not always with a published, validated scale. According to the findings, NSS can be conceptualized as one of the core features of schizophrenia. Hence, we may hypothesize NSS to be the consequence of the genetic liability towards the disease. NSS may serve as surrogate markers of the schizophrenic disease process with higher scores corresponding to a trait-like liability.

## 8. Limitations

The study had various limitations. It was conducted in a tertiary care hospital setting where mainly assessment of severely ill patients is done and the sample size was modest. Females were not inducted in the study which is a primary limitation in the applicability of the present findings to female schizophrenia patients. All of the patients in the study were right-handed; therefore, the impact of the cerebral dominance could not be studied. The examiner for NSS was not blinded regarding FRS.

## 9. Conclusions

The correlations between NSS and clinical symptoms in the present study were relatively modest but significant. The correlation coefficients between NSS total and total positive and negative symptoms scores show a weak relation between NSS and positive symptom severity. Hence, our results confirm previous studies demonstrating that NSS are more prominent in patients with negative symptoms than in those with positive symptoms. NSS may serve as surrogate markers of the schizophrenic disease process with higher scores corresponding to a trait-like liability.

## Figures and Tables

**Table 1:** Comparison of sociodemographic variables (continuous variable: age) between patients of schizophrenia with and without first rank symptoms (FRS), their first degree relatives (FDR), and normal control.

	Schizophrenia with FRS (mean ± SD) *N* = 30	FDR of schizophrenia patients with FRS (mean ± SD) *N* = 30	Schizophrenia without FRS (mean ± SD) *N* = 30	FDR of schizophrenia patients without FRS (mean ± SD) *N* = 30	Normal controls (mean ± SD) *N* = 30	*F* (df)	*P*
Age	30.25 ± 7.86	35.30 ± 9.77	30.13 ± 7.14	34.33 ± 10.07	34.20 ± 8.53	2.37 (4,145)	0.056
Income	2383.30 ± 3188.57	4283.30 ± 2875.83	900.00 ± 1743.95	5033.30 ± 3652.90	5250.00 ± 3899.04	10.502 (4,145)	0.001

SD: standard deviation.

**Table 2:** Comparison of sociodemographic characteristics (categorical variables: residence and employment) between patients of schizophrenia with and without first rank symptoms (FRS).

Variable	Schizophrenia with FRS *N* = 30 *n* (%)	Schizophrenia without FRS *N* = 30 *n* (%)	*x* ^2^	df	*P*
Residence					
Rural	19 (63.3%)	24 (80.0%)	2.058	2	0.357
Semiurban	9 (30.0 %)	5 (35.7 %)			
Urban	2 (6.7%)	1 (3.3%)			
Employment					
Employed	16 (53.3%)	13 (43.3%)	40.397	4	0.0001
Unemployed	14 (46.7%)	17 (56.7%)			
Religion					
Hindu	23 (76.7%)	24 (80.0 %)	2.390	8	0.967
Muslims	2 (6.7%)	3 (10.0 %)			
Others	5 (16.7%)	3 (10.0 %)			
Ethnicity					
Tribal	5 (16.7 %)	4 (13.3%)	0.320	4	0.989
Nontribal	25 (83.3%)	26 (86.7%)			
Marital status					
Single	11 (36.7%)	17 (56.7%)	2.411	1	0.121
Married	19 (63.3%)	13 (43.3%)			
Family history					
Absent	28 (93.3%)	27 (90%)	—	—	—
Schizophrenia	1 (3.3%)	3 (10%)			
Mood disorder	1 (3.3%)	0 (0%)			

**Table 3:** Comparison of age of onset of psychosis and duration of untreated psychosis (DUP), Scale for the Assessment of Positive Symptoms (SAPS) scores, and Scale for the Assessment of Negative Symptoms (SANS) scores between schizophrenia patients with and without first rank symptoms.

Variables	Schizophrenia with FRS (mean ± SD)	Schizophrenia without FRS (mean ± SD)	*t* (df) *F*	*P*
Age of onset of psychosis	28.52 (6.69)	25.80 (6.63)	1.58 (58) 0.093	0.762
DUP (in months)	19.85 (36.26)	11.33 (23.03)	1.09 (58) 2.220	0.142

Scale for the Assessment of Positive Symptoms	Schizophrenia with FRS (mean rank)	Schizophrenia without FRS (mean rank)	Mann-Whitney *U* test	*P*

Global rating of severity of hallucinations	40.27	20.73	157.00	0.0001***
Global rating of delusion	32.62	28.38	386.50	0.336
Global rating of bizarre behaviour	28.27	32.73	383.00	0.294
Global rating of FTD	25.27	35.73	293.00	0.003**
Total SAPS score	36.07	24.93	283.00	0.013*

Scale for the Assessment of Negative symptoms	Schizophrenia with FRS (mean rank)	Schizophrenia without FRS (mean rank)	Mann-Whitney *U* test	*P*

Global rating of affective flattening	26.37	34.63	326.00	0.050
Global rating of alogia	28.07	32.93	377.00	0.237
General rating of avolition and apathy	26.37	34.63	326.00	0.054
Global rating of anhedonia and asociality	27.35	33.65	355.50	0.147
Global rating of attention	25.30	35.70	294.00	0.015*
Total SANS score	23.52	37.48	240.50	0.002**

*P < 0.05, **P < 0.01, and ***P < 0.001.

**Table 4:** Comparison of neurological soft sign (NSS) scores between patients of schizophrenia with and without first rank symptoms using Kruskal-Wallis test.

	*N* = 150 (mean ± SD)	Schiz. with FRS (A)	FDR of schiz. patients with FRS (B)	Schiz. without FRS (C)	FDR of schiz. patients without FRS (D)	Normal controls (E)	*χ* ^2^	*P* (post hoc)
Total abnormality score	1.52 ± 2.31	99.00	57.08	98.92	68.75	53.75	39.424	0.0001*** (A > B, D, E; C > B, D, E; D > E)
Filtered abnormality score	1.40 ± 2.16	98.65	58.20	99.77	69.48	51.40	41.852	0.0001*** (A > B, E; C > D, E; D > E)
Soft signs	1.19 ± 1.99	100.38	57.12	99.38	68.15	52.47	45.594	0.0001*** (A > B, D, E; C > B, D, E; D > E)
Hard signs	0.29 ± 0.80	81.68	68.87	91.25	71.70	64.00	19.309	0.001*** (A > B, E; C > D, E)
Filtered hard signs	0.11 ± 0.35	76.13	70.97	83.30	76.13	70.97	6.425	0.170
Motor domain	0.16 ± 0.58	80.60	72.83	80.60	75.47	68.00	6.774	0.148
Involuntary movements	0.15 ± 0.50	76.70	73.92	81.62	76.27	69.00	5.625	0.229
Muscle power	0.01 ±0.12	74.50	74.50	74.50	74.50	79.50	8.054	0.090
Muscle tone	0.01 ± 0.14	74.00	74.00	81.50	74.00	74.00	12.163	0.016
Cranial nerves	0.02 ± 0.45	78.98	71.30	89.10	71.68	66.43	12.661	0.013** A > E; C > D, E
Cognitive functions	0.18 ± 1.94	94.47	58.50	95.62	68.10	60.82	39.221	0.0001*** (A > B, E; C > D, E)
Reflexes	0.67 ± 0.33	84.00	69.00	86.50	71.50	66.50	16.681	0.002** (A > B, E; C > D, E)
Sensory	0.12 ± 0.79	82.25	71.28	88.30	69.17	66.50	17.389	0.002** (A > B, E; C > D, E)

*P < 0.05, **P < 0.01, and ***P < 0.001.

Schiz.: schizophrenia; FDR: first degree relative; FRS: first rank symptoms.

**Table 5:** Spearman Rho correlation between Total Scale for the Assessment of Positive Symptoms (SAPS) and SANS Scale for the Assessment of Negative Symptoms (SANS) and neurological soft signs of schizophrenia with and without first rank symptoms scores.

	Total SAPS score	Total SANS score
Total abnormality score (0–125)	−0.075 (0.567)	0.167 (0.203)
Soft signs	−0.075 (0.567)	0.150 (0.251)
Mirror movements	0.059 (0.654)	0.105 (0.426)
Cranial nerves	0.035 (0.790)	0.250 (0.054)
Domain II: cognitive functions	0.004 (0.973)	0.030 (0.817)
Domain III: reflexes	−0.078 (0.554)	0.348 (0.006)**
Domain IV: sensory	−0.107 (0.416)	0.037 (0.777)

**Correlation is significant at the 0.01 level (2-tailed).

**Table 6:** Linear regression model of demographic and clinical variables of schizophrenia with and without first rank symptoms.

	Adjusted *R* square	*F* (df)	Beta	*t*	*P*
Age	0.21	2.62 (7.22)	0.19	0.23	0.816
Years of education			−0.20	−1.049	0.306
Age of onset of psychosis			4.26	2.156	0.042*
Duration of untreated psychosis in months			1.81	1.719	0.100
Total SAPS score			−0.46	−2.105	0.047*
Total SANS score			0.76	3.740	0.001***
Frequency of FRS			0.32	1.326	0.198

*P < 0.05,   **P < 0.01, and ***P < 0.001.
